# Geochemical Background and Baseline Values Determination and Spatial Distribution of Heavy Metal Pollution in Soils of the Andes Mountain Range (Cajamarca-Huancavelica, Peru)

**DOI:** 10.3390/ijerph14080859

**Published:** 2017-07-31

**Authors:** Fernando Santos-Francés, Antonio Martínez-Graña, Pilar Alonso Rojo, Antonio García Sánchez

**Affiliations:** 1 Department of Soil Sciences, Faculty of Agricultural & Environmental Sciences, University of Salamanca, Avenue Filiberto Villalobos, 119, 37007 Salamanca, Spain; fsantos@usal.es (F.S.-F.); palrojo@usal.es (P.A.R.); 2 Department of Geology, Faculty of Sciences, University of Salamanca, Plaza de la Merced s/n., 37008 Salamanca, Spain; 3 Department of Environmental Geochemistry, Institute of Natural Resources and Agrobiology—IRNASA (C.S.I.C.), Calle Cordel de Merinas 40, 37008 Salamanca, Spain; antonio.gsanchez@irnasa.csic.es

**Keywords:** pollution soil, heavy metals, spatial distribution, geochemical background, environmental quality, kriging, Andes Mountain Range of Peru

## Abstract

Concentrations of seven heavy metals (Cd, Cr, Cu, Hg, Ni, Pb, and Zn) and one metalloid (As) as well as various parameters (pH, organic carbon, granulometric analysis and cation exchange capacity) were analyzed in 77 soil samples collected in the mining areas of La Zanja and Colquirrumi (Department of Cajamarca) and Julcani (Department of Huancavelica). Our study proposed geochemical baseline values for heavy metals in a natural region (La Zanja) from samples collected during the period of the environmental impact study (2006), that is, from an earlier period which occurred at the beginning of the exploitation of the current gold mine. The baseline values obtained were as follows: 8.26 mg·kg^−1^ for Cr; 56.97 mg·kg^−1^ for Ni; 22, 20 mg·kg^−1^ for the Cu; 47.42 mg·kg^−1^ for Zn; 27.50 mg·kg^−1^ for As; 4.36 mg·kg^−1^ for Cd; 4.89 mg·kg^−1^ for Hg, and 44.87 mg·kg^−1^ for Pb. Through the use of different indices of heavy metal contamination (geo-accumulation index (I_geo_), improved Nemerow index (I_IN_) and potential ecological risk index (RI)), the degree of pollution caused by mining activities in two areas, Colquirrumi and Julcani, which have a high density of mining sites in operation, was determined. The values obtained from these indices indicated that the Colquirrumi region was the most contaminated, followed by Julcani. The area of La Zanja, despite being free of mining operations, presented slight diffuse pollution. Several positive correlations were obtained, with a high level of significance, between pH, organic carbon content, cation exchange capacity, and the Cr, Pb and Ni concentrations of the soils. The spatial distribution of the heavy metals was realized by means of the interpolation method of ordinary kriging. The results obtained and the experience gained in this work were necessary to facilitate the identification of soil contamination processes in high altitude areas of the Andes Western Cordillera (Peru) as a basis for taking appropriate measures when restoring soils, during mine closure processes, and to protect the quality of soil resources.

## 1. Introduction

Heavy metals in soils have been identified as essential components of the environment and the food chain and as important factors inhuman health [1,2,3,4,5,6]. The term geochemical baseline—officially presented in 1993 under the International Geological Correlation Program as the Global Geochemical Baselines—refers to the natural variation in the concentration of an element in the surface environment, at a determined place and time. This concept includes natural geographic concentrations (background level) and the diffuse anthropogenic contribution in soils.

Background level is a measure that is used to differentiate between the concentration of the natural compound and the concentrations with an anthropogenic influence in a given environmental sample [7,8]. The concentrations of heavy metals in the natural soil background depend on the geological substrates and the processes that form the soils [9,10]. Rocks have a large influence on the content of heavy metals in soils, with concentrations sometimes above critical values [11]. However, it is almost impossible to establish natural background levels, i.e., the geochemical composition of virgin soils, since atmospheric deposition can contaminate soils with certain trace elements [12,13]. The calculation of the geochemical baseline is therefore more useful, since it represents conditions where a certain human impact on the environment already exists [14,15,16]. The soil compartment receives significant amounts of contaminants from different sources each year. Therefore, this compartment acts as a sink for a wide variety of emissions comprising several heavy metals, some of which are toxic. Sources of heavy metals in the environment include aerial deposition of particles emitted by different human activities, including mining.

The calculation of environmental geochemical baselines is necessary to assess the current state of the environment and to provide guidelines and quality standards in environmental legislation and policy-making, especially in the evaluation of contaminated soils and in environmental risk assessment [17]. Numerous studies have been carried out in different regions worldwide to estimate the geochemical background concentration and the baseline of heavy metals, and there are two main methods used to estimate the background level: direct (geochemical), and indirect (statistical). In this study, we followed the direct method, which uses samples not affected by industrial or mining activities, or samples from relatively pristine sites. This method generally uses simple statistical values, such as the median or the mean, to estimate background concentrations [18,19]. A geochemical baseline should be determined separately for each heavy metal in geologically different regions, otherwise the limit values for contaminated soils may be lower than the natural concentrations (background levels) calculated for an extensive area [20]. As a result, the soil trace element content is very variable, which makes the use of normative values of environmental legislation of other countries or regions inappropriate, as they must be determined locally. Currently, published studies that have determined the concentrations and patterns of spatial distribution of heavy metals in soils located at high altitudes in the Peruvian Andes are extremely limited.

The analysis of the environmental impacts of a mining activity is made through the difference between the situation of the environment before the activity was conducted, and after the development and cessation of mining activity. The investigation of the baseline of a territory represents a measure of the geochemical variations of its surface formations (rocks and soils) and is considered of great interest, not only from a scientific and mining point of view, but also constitutes a very important tool for environmental planning, environmental health, and sustainable development policies worldwide [21]. If an exploration campaign is successful (i.e., the location of an economic mineralized body has been discovered), at that time, the mining company must begin an investigation of the environmental baseline. This allows the development of a frame of reference to be able to properly control the environmental changes generated during and after the mining activity. To do this, baseline research has to be conducted before the activity in question has significantly affected the environment. This was the case of our study of the mining project of La Zanja (Cajamarca), which was carried out in 2006, during the period of the Environmental Impact Study and prior to the beginning of mineral extraction.

The first step in a baseline study is to define the thresholds of toxicity for the different pollutants, where the normal values of potentially polluting substances present in natural soils without human influence (known as the background level) are calculated, which corresponds to the normal value of an element in a given environment [22,23]. The calculation of the background level is often a complicated task since soils without any type of contamination are almost impossible to find due to atmospheric deposition of long distance trace elements and human activity [19]. Given these difficulties, the baseline values should show an average value and a range of concentrations of heavy metals for a specific area and at a specific time, as well as considering the diffuse entry of these elements into soils [24]. The value of the baseline should correspond to a statistically significant deviation from the addition to the arithmetic or geometric mean twice the value of the standard deviation for each element studied [25]. From the identification of the geochemical baseline values of a region, soil quality standards can be established (e.g., reference and intervention levels). The current methodology for assessing environmental quality through the content of metalloid and heavy metals in the soil includes the calculation of several pollution indices such as they geo-accumulation index (I_geo_), the potential ecological risk index (RI), the improved Nemerow index (I_IN_), etc., of the surface horizons of soils.

In this study, a geostatistical approach was adopted to calculate the spatial distribution of the geochemical anomalies of this region of the Peruvian Andes, specifically, the spatial distribution of the concentrations of seven heavy metals and one metalloid (Cu, Zn, Pb, Cr, Cd, Ni, As, and Hg) in soils using the Inverse Distance Weighting (IDW) and ordinary Kriging interpolation methods [26]. The spatial variability of heavy metal concentrations in soils contains basic information used to identify possible sources of contamination [27], to control and evaluate environmental risks [28], and outline the remediation strategies of the place. Considering the cost of extensive and repeated soil sampling and analysis is impractical and mapping with the spatial distribution of soil contamination requires spatial interpolation methods; consequently, interpolation techniques such as kriging are widely used in the investigation of contaminated soils [29,30,31].

Studies of soil contamination by heavy metals need to focus on identifying areas with a high risk of contamination. Samples from these types of areas usually have atypical spatial values at the local level [32]. The fundamental pollution problems (natural and/or anthropic) in the watersheds studied in Cajamarca and Huancavelica (Peru) are mainly derived from the removal of heavy metals from alteration zones and mining operations located at high altitude. During the rainy season, these materials go directly to the lower agricultural valleys and to the main river flows of this region. Therefore, it was considered necessary to conduct a heavy metal investigation, both in the soils and in the surface waters of these mining areas to provide baseline information on the impact of anthropogenic environmental pollution. Furthermore, the generation of acidic waters has been recognized as an important problem of environmental pollution in the last three decades [33].

The main objective of this study was: (1) to analyze the concentrations of eight heavy metals (Cr, Ni, Zn, Cu, Pb, As, Cd, and Hg) to calculate the background level in three mining areas of the provinces of Cajamarca and Huancavelica (La Zanja, Colquirrumi and Julcani) and calculate the geochemical baseline in La Zanja; (2) for purposes of comparison, to calculate several indices of heavy metal contamination (I_geo_, I_IN_, and RI) in the superficial horizons of soils; (3) to obtain geochemical maps revealing the degree of contamination of soils by heavy metals; and (4) to evaluate the correlations between heavy metal concentrations and soil properties (pH, organic carbon, cation exchange capacity, etc.). This environmental research study aimed to understand the influence of mining activity on the environmental contamination of high altitude areas within the provinces of Cajamarca (3200–3900 m) and Huancavelica (4100–4400 m) in the Western Cordillera de Los Andes (Peru). The results of this study will contribute to the creation of an environmental database of the soils and waters of the Cajamarca and Huancavelica regions (Peru), which will provide the authorities with monitoring data on the pollution caused by mining activities and will assist in the development of appropriate management strategies to control the contamination of heavy metals in soils and waters.

## 2. Materials and Methods

### 2.1. Study Area

**Figure 1:**
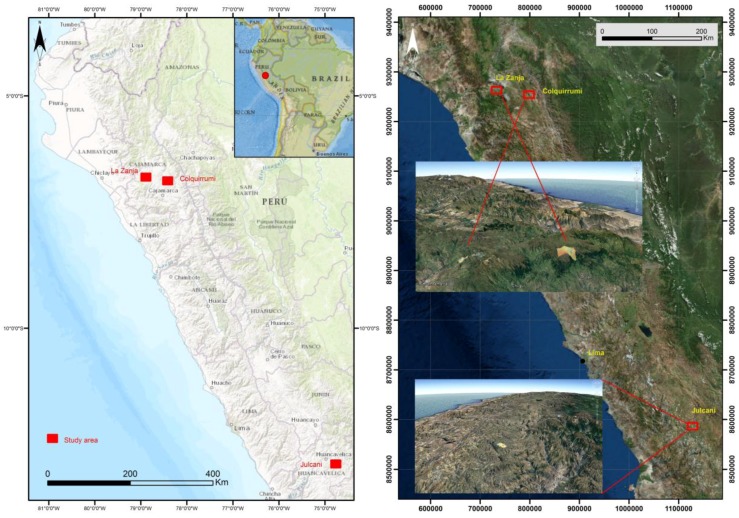
Situation of the study area: Cajamarca and Huancavelica.

The department of Cajamarca is in the extreme north of Peru, in the northern zone of the Western Cordillera de los Andes. It is limited in the North with the neighboring country of Ecuador, the South with the Department La Libertad, the East with the Department of Amazonas, and the West with the Departments Piura and Lambayeque (Figure 1). The most important border of the Department of Cajamarca is marked towards the east by the river basin of the Marañón that separates it from the Department of Amazonas.

The mining site of Colquirrumi is in the district of Hualgáyoc (Cajamarca), 88 km north of the city of Cajamarca and south-west of the district of Bambamarca. The La Zanja Project is in the village of La Zanja (also called La Redonda), in the district of Pulán, Province of Santa Cruz de Succhabamba, Department of Cajamarca. The Department of Huancavelica is in the south-central zone of the Western Cordillera of the Andes. It has borders in the North with the Department of Junín, in the East with Ayacucho, the South with the Departments of Ica and Ayacucho, and in the West with Ica and Lima. The mining site of Julcani is in Angaraes Province (Huancavelica), District of Cochaccasa.

The Departments of Cajamarca and Huancavelica are located at high altitudes above sea level (between 3200–4400 m), with very rainy climates (between 800–1300 mm) and cold temperatures (average temperature of the year between 5–13 °C), with an evapotranspiration between 900–1140 mm. The climate is sub-humid, with seasonal rains and frequent periods of drought. Seasons of intense rain occur between the months of November and April. In the upper parts of the mountain range, temperatures drop below 0 °C.

From a geological perspective, the mining area of Colquirrumi-Sinchao is constituted by folded sedimentary rocks from the Cretaceous period that have been improved by stocks, dykes and sills composed of diorite and granodiorite of the Miocene Half-Superior. All these bodies are located within a NW-SE regional course that includes the well-known mineral deposits of Colquirrumi, Hualgayoc, San Agustín, Shinchao, Constancia, Cerro San José, Cerro Corona, Minas Congas, Galeno, Michiquillay-Lambayeque, Mansita, San Agustín, Tres Cruces, etc.

The mineral deposits of the Hualgáyoc district are of different types: veins, mantles, bodies and porphyries with disseminated mineralization, and in stockwork which contain complex ores of Ag-Zn-Pb (Colquirrumi) and Cu-Ag-Au-Zn (Sinchao) from meso to epithermal. The Colquirrumi-Sinchao deposit is a replacement in the form of limestone and sandstones, which are covered with volcanic materials. Towards the NE appears a stock of medium-fine grain diorite and quartz-diorite porphyries. The igneous activity continues with the location of dacite domes and volcanic rhyolites and/or dacites that partially cover the other units. The final phase consists of rhyolitic dikes that cut epithermal mineralization. The ore is composed of chalcopyrite, tetrahedrite, galena, sphalerite and as gangue pyrite, quartz, chalcedony, and iron oxides [34,35]. In Hualgayoc and Julcani, there have been clearings, dumps and other liabilities as a result of decades of past mining that are currently unrecovered.

**Figure 2:**
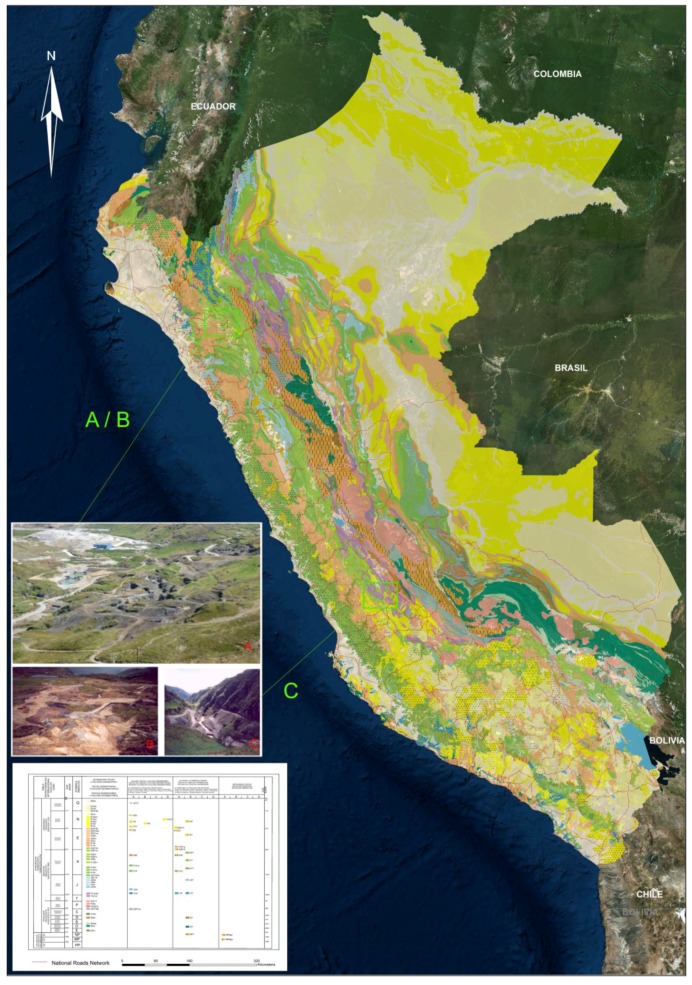
Geological context and mining areas with clearing of Colquirrumi (**A**); clearing of the Maruja, Tres Mosqueteros, Constanza and Cleopatra mines in La Zanja (**B**); and Julcani clearing in the Tingo creek (**C**).

In the deposit of the Zanja are rocks of volcanoclastic origin, consisting of a sequence of tuffs, tuffs and lavas, of andesitic, dacitic, and rhyolitic nature, belonging to the Llama, Porculla and Volcanic Huambo formations (Figure 2). The geological ages of these rocks vary from the Upper Eocene to the Upper Miocene and Late Pliocene. Near the project area, there are also subvolcanic bodies associated with a volcanic-magmatic event contemporaneous with pyroclastic deposits. Cretaceous sedimentary rocks belonging to the Goyllarisquizga group, strongly folded and faulted, are in an inward position and discordant to previous sequences. On the volcanoclastic sequence, and influenced by the subvolcanic bodies, minerals of economic value (Au and Ag) have been identified, as was the case of San Pedro Sur and Pampa Verde, which corresponded to epithermal processes of high sulfidation. This type of deposit is characterized by a clearly zoned hydrothermal alteration, with the presence of silicification in the central part and a gradation to argillic rocks (quartz-alunite-dickite, quartz-kaolinite, and illite-smectite-kaolinite-sericite) towards the edges.

The studied area of Julcani was geologically formed by a series of sedimentary rocks of Paleozoic-Mesozoic age including: (1) phyllites and sandstones of lower Devonian age (Excelsior Group), where these rocks are strongly deformed in syncline folds and anticline orientations NW; (2) a sequence of conglomerates, sandstones and shales, known as red layers (Mitu Group) that overlie, in erosive discordance, the metamorphosed and folded rocks of the Excelsior Group from the Permian period; (3) limestones and sandstones (Pucará Group) that usually contain abundant chert from the Triassic-Jurassic age; and (4) a volcanic sequence that occurred in the surroundings of Julcani comprised of dacites to rhyolites in a grouping of volcanic centers orientation WNW-ESE (Julcani Formation), and these volcanic materials have been dated to 10.4 Ma (upper Miocene).

In Julcani, deposits of economic value have been identified (including Au, Ag and polymetals, Cu, Pb) in mineralization located and genetically related to the Julcani volcanic center, which comprises a grouping of volcanic dacitic centers to the WNW-ESE, composed of pyroclastic rocks, lavas, endogenous domes and dikes. It is a filonian type deposit of fracture fillings with metallic content of Ag, Pb, Cu in the form of a system of irregular veins with bonanza bodies and gaps placed in pyroclastic rocks of the Julcani Formation. Most deposits in this mining district are within a volcanic sequence (Miocene), among them we have the following mines: Herminia, Mimosa, Sacramento, Estela, Temtadora, Nuestra Señora del Carmen, Rita, Achillia, etc. Another mineralized zone is of phyllites (Lower Devonian), where the mines are the Pucará, Bernabé and Contaglapampa [36].

### 2.2. Soil Samples and Analysis

The morphological inventory and the sampling of the soils has been realized by considering all units of soils that have been developed based on different types of rocks that exist in this territory. Thus, the selection of sampling points of the soil profiles has been based on lithological criterion, since in natural soils, the heavy metals present are inherited directly from the parent rock. In addition, the content of heavy metals (Pb, Cu, Zn, Ni, Cd, As, Hg, and Cr) produced by primary dispersion caused by the mineralization of the parent rock (background level) has been characterized, independently of that produced for mining activity, for this purpose, all horizons (A- and B- and subsurface-C or R-) of the soils were sampled. The sampling strategy focused specifically on the surface horizons (A and B) and deep or bedrock (C or R); the latter being unlikely to be contaminated by atmospheric deposition.

Four sub-samples of replicate soils were randomly collected at each sampling point within a 1.5 m × 1.5 m grid and mixed to obtain a composite sample of about 750 g in weight. In this study, a total of 77 soil samples were collected (77 × 4 subsamples = 308). Samples were air-dried and sieved through a 2 mm mesh. The determination of the physical and chemical properties was carried out as per traditional methods of soil analysis: organic matter by oxidation with potassium dichromate, granulometric analysis using the Robinson pipette method, pH (water 1:1) and capacity cation exchange (CEC) using the ammonium acetate method. The total contents of heavy metals in soil were analyzed as per the procedure recommended by the European Union International Organization for Standardization (ISO) standard 11466. Extraction was performed with a mixture of nitric acid and hydrochloric acid in a microwave oven, with determination by inductively coupled plasma mass spectrometry (ICP-MS) (Elan 6000, Perkin-Elmer, Waltham, MA, USA). The analysis was carried out by the Chemical Analysis Service of the University of Salamanca through the digestion of samples in a microwave oven (Ethos Plus Microwave Lastation, Milestone Inc., Shelton, CT, USA), using the standard USEPA method 3052. To calibrate the equipment, standard solutions (panreac) of 1000 mg/L of all metals were used, which were calibrated from 10–100 ppb.

### 2.3. Statistical Analysis Samples

SPSS software v.23.0 (IBM, Armonk, NY, USA) was used for statistical analysis. The results have allowed us to obtain different values of interest: arithmetic mean, geometric mean, median, range, standard deviation, coefficient of the variable, kurtosis, correlation analysis between heavy metals and soil properties. In addition, various pollution indices have been calculated in order to determine the levels of contamination of heavy metals in soils. The geographic information system (GIS) analysis (ArcGis v10.4, Esri, Redlands, CA, USA) with extensions Spatial analysis tools and geostatistical analyst has calculated the degree of spatial variability of each heavy metal using ordinary kriging [37]. Kriging is an advanced geostatistical procedure that generates a surface estimated from a set of dispersed values, generating an interactive investigation of the spatial behavior, in our case of soil contamination by heavy metals. Unlike the deterministic interpolation methods IDW and Spline (which depend directly on the surrounding measured values, which determine the smoothness of the resulting surface), the geostatistical kriging interpolation method is based on statistical models that include autocorrelation, i.e., The statistical relationships between the measured points. In the evaluation of soil contamination, this technique of geographic statistics generates a prediction surface of the distribution of heavy metals, providing certainty or precision in the prediction. For the creation of the prediction surface map, it uses an equation (Equation (1)) that discovers the rules of dependence between points and then performs the prediction of spatial or geographical distribution.

(1) $$Z\;\left(S_{0}\right)=\overset{N}{\underset{i=1}{\sum }}\lambda_{i}Z\;\left(S_{i}\right)$$

where (*S_i_*) = the measured value at the location *i*; *λ_i_* = an unknown weight for the measured value at location *i*; *S*_0_ = the location of the prediction; and *N* = the number of measured values.

In this work, the applied kriging method was the ordinary one which assumes that the distance or direction between the sample points reflects a spatial correlation that can be used to explain the surface variation.

## 3. Results and Discussion

### 3.1. Soil Study

**Figure 3:**
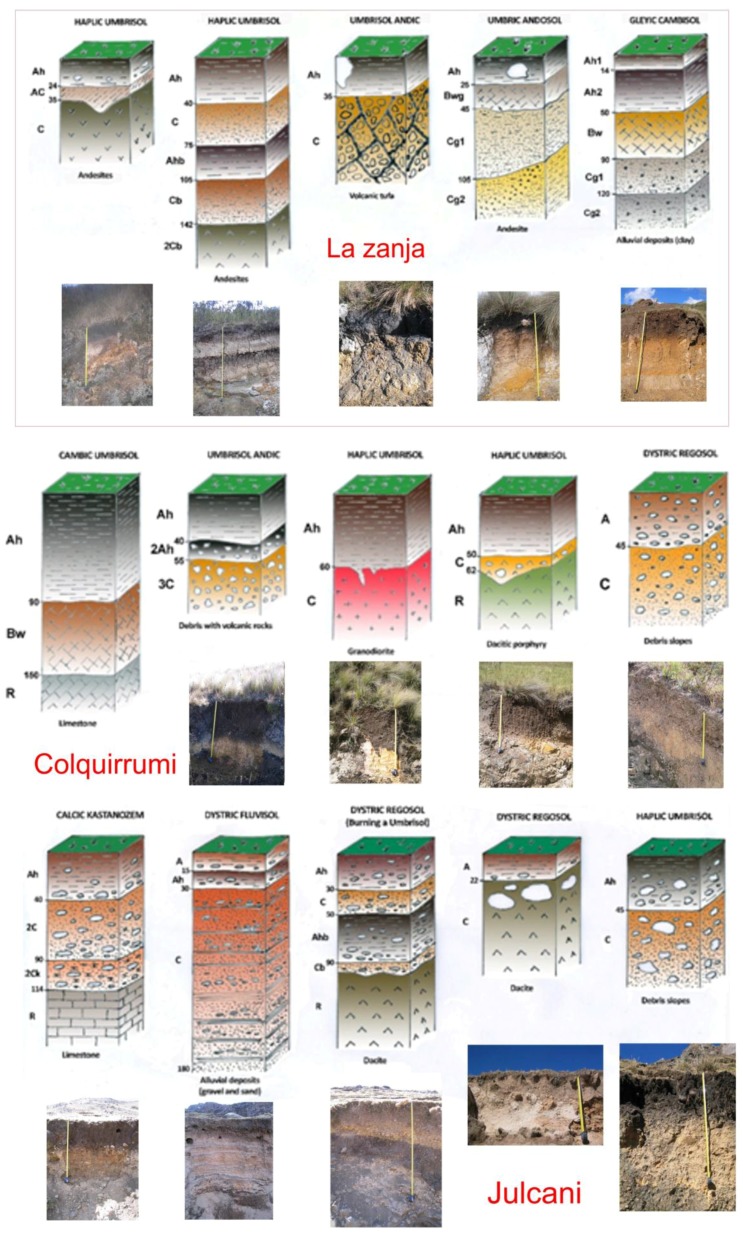
Morphology of the soils most representative of the studied areas.

The predominant soils in these sectors located at high altitudes of the Andes Mountains are of little development, being located in steep areas with strong slopes (Figure 3). Most soils have been classified as Haplic Umbrisols (hyperdystrics and andics), which are acidic soils (average value of pH = 4.7) that have developed on volcanic rocks and are constituted by a surface horizon (Horizon A) that is very dark to black and high in organic matter (average content of 16.44%), with a low degree of saturation in bases (mean value = 9.5%). Some of these umbrisols have Andean properties (low bulk density and thixotropy) and can even be classified as umbric andosols.

The Andes mountain range is subject to intense erosive processes that increase sediment and soil trawling to the bottoms of valleys or depressions. It is in these zones where erosion processes dominate over edaphic processes, where soils have small development (Dystric Regosols and Gleyic Cambisols) and are developed on sloping landslides. These soils differ from the previous ones in that the superficial horizon has a low or moderate content of organic matter (4.65%) and, therefore, a brown color. Cambisols are characterized by having a subsurface horizon (Bw), of alteration (horizon of diagnostic cambium), and are brownish-gray or yellowish-brown color.

In the studied area, rocky outcrops that are associated to soils with little thickness with the denomination of Umbric Leptosols are also frequent, and limestone rocks have been observed with Rendzic Leptosols and Calcic Kastonozems. In some areas, there are small depressions where water accumulates (bofedal), and the reducing environment favors the accumulation of organic matter, giving rise to Fibric Hemic and Sapric Histosols (Pebbles). In the “valley bottoms” originating from rivers, streams and streams that run through the studied area, recent alluvial sediments have been deposited, consisting of gravel and sand with a thickness to the order of two meters. The soils that have developed in the current channel of streams and rivers are classified as Dystric Fluvisols.

A classification of these soils has been made by considering their morphology and analytical data, by the taxonomy of the World Reference Base (WRB). The statistical data of the main studied properties of the soils of the Zanja, Colquirrumi, and Julcani are presented in Table 1. In the analyzed samples, the physical and chemical properties of the soils have been discarded those that are compact and hard of some horizons C and R, giving a total of 59 samples analyzed.

### 3.2. Concentration of Heavy Metals in Soils of La Zanja, Colquirrumi, and Julcani

In this study, our aim was to understand the natural contents of the soils (pedogeochemical background) to detect the intensity of the contamination of the soils and to compare the results with norms or regulations of other countries worldwide [38,39].

In the statistical analysis of this study, the data of the most superficial horizons (A and B) and underlying (C) horizons were treated separately. The more superficial horizons or solum were those that provided information on the levels of pollution caused by the processes of soil formation and by anthropogenic sources, whereas the underlying horizon (bedrock) exclusively represents the lithogenic contributions, since there is little probability of contamination through atmospheric deposition [40]. It is for this reason that samples of soil bedrock were used to determine the level of the natural geological background.

The average values of the heavy metals obtained from the statistical analysis of the soils of La Zanja can be seen in Table 2, where other bibliographical data of levels obtained in other countries have also been attached for purposes of comparison.

Background levels of the Ni, Cu, Hg, As, and Pb metals were above the world average. The Zn content was equivalent to the world average, and the concentration of Cr was lower than the world average. The highlight of the mining area of La Zanja was the high content of Cd soils, since it had a value higher than the world's top ranking sample.

As for the values obtained from the Colquirrumi soils, it is worth noting the abnormally high values across all samples for Pb content, where the background level was 1539 mg·kg^−1^, compared to the world average of 15 mg·kg^−1^ and a global range between 2 and 200 mg·kg^−1^ [10], although the range has reached as high as 1500 mg·kg^−1^ [42]. Obtaining such high values in Pb content forced us to repeat the sample analysis several times, given the incredulity regarding the resulting high values. In contrast, Cr metal had values considered inferior to the background levels reported in other parts of the world. Regarding the Ni content, the background level was lower (12.05 mg·kg^−1^) when compared to the world average. Furthermore, in numerous soil samples, Cu content exceeded the values of the world average (12 mg·kg^−1^), and in some soils Cu values reached between 100 and 500 mg·kg^−1^. As for Zn, the background level (165.6 mg·kg^−1^) obtained in this study exceeded the world average values of 40 mg·kg^−1^. In some soil samples, values higher than 3000 mg·kg^−1^ were reached. Of the soil samples collected in Colquirrumi, only three did not exceed the world average (20 mg·kg^−1^) regarding As content, where the background level (218.1 mg·kg^−1^) was up to 11 times higher than most other countries in the world, although the range reached as high 250 mg·kg^−1^. Large As abnormalities have been observed in some soil samples, with values to the order of 1000 mg·kg^−1^. Finally, it was noted that the values of the background levels of Cd and Hg were high, to the order of 15 and 70 times above the world averages. To summarize, from the study of heavy metals in soils in the mining area of Colquirrumi, we must highlight the geochemical anomalies in Pb, As, Cd, Hg, Cu, and Zn.

In relation to the values obtained in the soils of Julcani, it is necessary to point out the following: Cr, Ni, and Cu metals presented background and reference levels considered inferior to those described in the literature regarding other parts of the world. None of our soil samples exceeded the world average values, in terms of the Cr content. For Ni content, only two soil samples exceeded the values of the world average fund level (25 mg·kg^−1^). However, the concentrations of Zn and As were slightly higher than those obtained in other countries. The background levels of Pb, Cd, and Hg obtained from Julcani were 13, 14, and 34 times higher, respectively, than the levels obtained in soils from other parts of the world.

#### 3.2.1. Box and Whisker Plots of Heavy Metal Concentrations in La Zanja, Colquirrumi and Julcani

**Figure 4:**
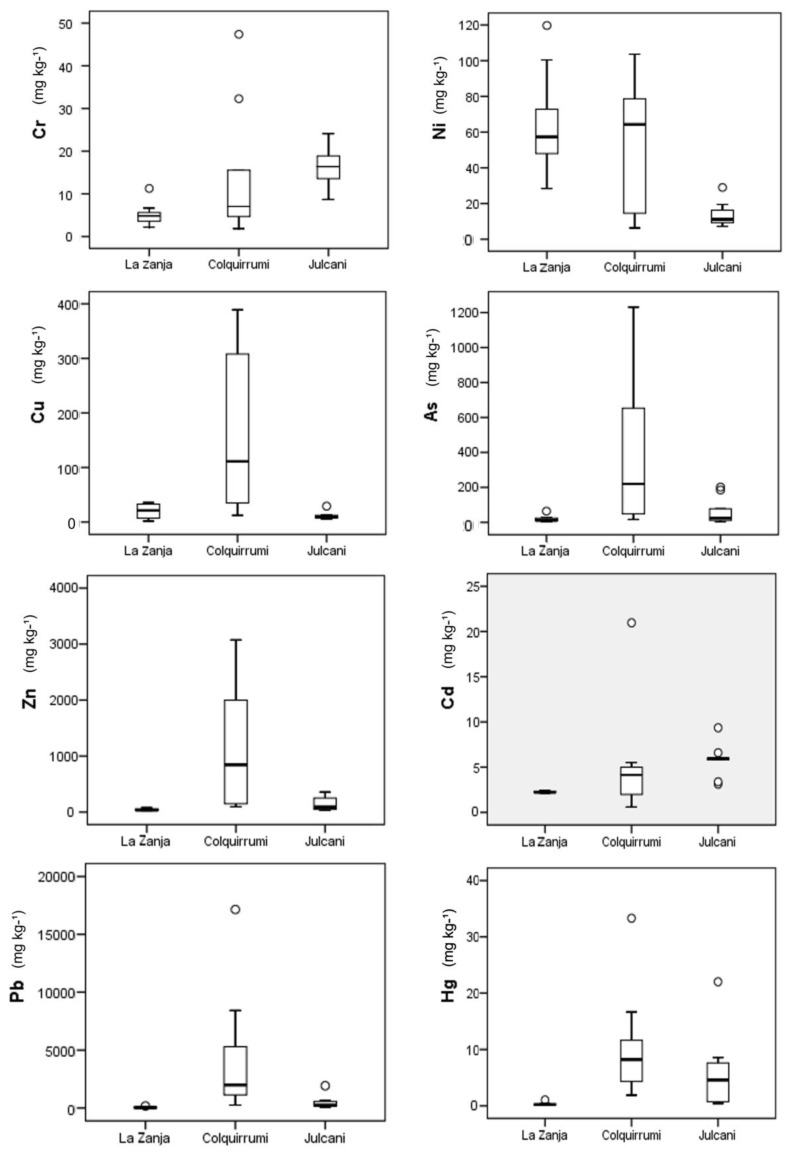
Box and whisker plots of heavy metal concentrations.

The box and whisker plots in Figure 4 show a summary of the basic statistics of the concentrations of heavy metals studied in the soils of the Western Cordillera of the Peruvian Andes. When comparing the concentrations of heavy metals in the three areas studied, it can be described better graphically through several box plots. In La Zanja the contents were lower with respect to Colquirrumi and Julcani, which was justified by the null or weak contamination of soils of La Zanja, given the lack of mining activities in the sampling year (2006) when compared to areas with extensive and ancient mining activities such as Colquitrumi and Julcani. Only the area of Julcani (Huancavelica) had lower Ni and Cu contents than La Zanja (Cajamarca) as both zones belong to two different metalogenic belts.

#### 3.2.2. Geochemical Baseline Study of the Soils of La Zanja

To calculate the geochemical baseline, the geometric mean was used with a normal logarithmic heavy metal distribution, and in cases where the variables were strongly asymmetric and their transformation could not be adjusted to normal, then the median was used [43]. The normal distribution of metal concentrations was investigated by performing the Kolmogorov-Sminov test, although this can also be inferred by the existence of high kurtosis values.

In the case of Zanja soils, concentrations of heavy metals from the surface horizons were used to calculate the baseline, which was conducted by adding the geometric mean to twice the standard deviation [25] (Table 3).

Soils studied from La Zanja were characterized by high baseline values for Ni, Cu, Pb, Cd, and Hg. The application of the regional geochemical baseline values proposed in this study will allow the rapid identification of sites that could be affected by pollution processes due to current mining exploitation in the area of La Zanja. In addition, this information will also be very useful in the future, when it is time to close the mine and implement a restoration plan to try to reproduce the environmental conditions that existed prior to mining.

### 3.3. Assessment of Environmental Risks: Pollution Rates

The state of heavy metal contamination in soils from the areas studied in this work were evaluated using different quantitative contamination rates.

#### 3.3.1. Pollution Factor and Nemerow Index

Using the pollution factor and Nemerow Index to determine pollution levels in the La Zanja area showed that most soil samples (70%) were contaminated, 20% were moderately contaminated, and 10% were strongly contaminated, mainly by Cr, As, and Pb. These data does not represent the reality observed on the land itself of the environmental conservation status of the territory, since La Zanja was a natural area without any type of mining when the samples were collected in 2006. Therefore, the soil samples obtained in this area were expected to appear low or very low on the contamination indices. These results question the validity of these indices to calculate the degree of soil contamination. In agricultural soils and in less industrialized areas of the Northern Plateau of Spain [44], the use of these pollution indices showed that the soils were between contaminated and heavily contaminated. That is, these results proved similar to those obtained in the Peruvian Andes that also did not adjust to the existing degree of contamination. As a result, with the experience already obtained, we verified that the assessment of environmental risks was better reflected reality by using other similar pollution indexes, such as the geo-accumulation index (I_geo_) and the improved Nemerow index (I_IN_).

#### 3.3.2. Geo-Accumulation Index and the Improved Nemerow Index 

The I_geo_ is calculated from equation (Equation (2)) [45]:

I_geo_ = log_2_(C_i_/1.5B_i_) (2)

where C_i_ is the measured concentration of the i metal examined in the soil, and B_i_ is the background level of the *i* metal. The factor 1.5 was used to correct possible variations in the background values of a particular metal in the environment. The I_geo_ for the eight heavy metals studied are summarized in Table 4. The I_geo_ of the analyzed heavy metals allowed the analysis of the single factor contamination index to evaluate the presence of each individual metal and its level of contamination in the study area.

However, the I_IN_ shows the degree of general pollution caused by the simultaneous presence of the nine heavy metals. In La Zanja, considering the I_geo_, most soils were included in Class 0 (I_geo_ ≤ 0) and Class 1 (0 < I_geo_ ≤ 1), that is, uncontaminated to slightly contaminated, with 67.7% and 17.8% of the samples, respectively, and only some soils in Class 2 (1 < I_geo_ ≤ 2), which were moderately contaminated by Cr, As, and Pb in 5.45% of the samples. Soils were not included in Classes 3, 4, 5, and 6 (from moderately contaminated to extremely contaminated). In Colquirrumi, samples of the soils studied were included in all classes ranging from uncontaminated to extremely contaminated soils: Class 0 (48.1% of the samples), Class 1 (16.3%), Class 2 (20.9%), Class 3 (5.4%), Class 4 (9.3%), Class 5 (0%), and Class 6 (16.3%). In Julcani, the soils, were classified as Class 0 (54.2%), Class 1 (30.5%, Class 2 (6.9%), Class 3 (5.6%), and Class 4 (1.4%), which ranged from uncontaminated to heavily contaminated.

By considering the I_geo_, the levels of contamination (from low to high) of the heavy metals in the superficial horizons of the soils was as follows: La Zanja < Julcani < Colquirrumi.

The I_IN_ was calculated by Equation (3):

(3) $$I_{\mathrm{IN}}=\;\surd \;½\;\left({I_{\mathrm{geomax}}}^{2}\;+\;{I_{\mathrm{geoave}}}^{2}\;\right)$$

where I_geo_max is the maximum I_geo_ value of all metals in a sample, and I_geo_ave is the arithmetic mean of the I_geo_. In La Zanja, when considering the I_IN_, the soils ranged from non-contaminated to moderately contaminated: 20% of samples were in Class 0 (I_IN_ < 0.5), 50% in Class 1 (0.5 ≤ I_IN_ < 1), and 30% of soils in Class 2 (1 ≤ I_IN_ < 2). However, it should be pointed out that the small degree of contamination indicated by this index may be due to anthropogenic processes (atmospheric deposition of metals, medium or long distance), which led to an increase in geological concentrations in the soils of La Zanja. The main external sources of heavy metals in the soils of this area are through diffuse pollution, and wet and dry deposition caused by metallic mining and the processing of minerals from bordering areas that has led to the accumulation over the long-term use of heavy metals. In Colquirrumi, all soil samples were classified as moderately contaminated Class 2 (33.3% of soil samples) to the highly contaminated Class 3 (2 ≤ I_IN_ < 3, 66.7% of the samples) based on the I_IN_. The soils of the Julcani area were considered as non-contaminated to highly contaminated: Class 0 (22.2% of the samples), Class 1 (22.2%), Class 2 (22.2%), and Class 3 (33.3%). By using the I_IN_, heavy metal contamination levels (from low to high) from soil surface horizons are as follows: La Zanja < Julcani < Colquirrumi.

#### 3.3.3. Potential Ecological Risk Index

The ecological risk index (E_r_^i^) evaluates the toxicity of trace elements in sediments and has been extensively applied to soils [46]. Soils contaminated by heavy metals can cause serious ecological risks and negatively impact human health due to various forms of interaction (agriculture, livestock, etc.) where highly toxic heavy metals can enter the food chain. Excessive accumulation of heavy metals in agricultural soils can affect the quality and safety of food and further increase the risk of serious diseases (cancer, kidney, liver damage, etc.), as well as impact ecosystems, thus combining environmental chemistry with biological toxicology and ecology [47].

To calculate the E_r_^i^ for individual metals, we used Equation (4):

E_r_^i^ = T_r_^i^ * C_f_^i^ (4)

where *Tr* is the toxicity coefficient of each metal whose standard values are Hg = 40, Cd = 30, As = 10, Co = 5, Cu = 5, Ni = 5, Pb = 5, Cr = 2, and Zn = 1 [48,49]; and C_f_^i^ is the contamination factor (C_f_^i^ = C_i_/B_i_), where *C_i_* is the measured concentration of the pollutant, and *B_i_* is the level of geological background.

To calculate the potential response rate to the toxicity of all the studied heavy metals (RI), we use Equation (5):

RI= ∑ ^m^_i=1_ E_r_^i^ (5)

The potential ecological risk index (RI) index reflects the general situation of pollution caused by the simultaneous presence of the eight heavy metals (Table 5).

Considering the individual ecological risk index (Er), the levels of heavy metal contamination of the surface horizons of La Zanja soils were considered to be of low contamination risk (Er < 40), with only one soil sample (14.3%) with a considerable risk of contamination (80 ≤ Er < 160) per Hg.

In Colquirrumi, a majority of the samples (87%) were considered to have a low risk of contamination (Er < 40); a small percentage of samples (4.2%) were considered to have a moderate contamination risk (40 ≤ Er < 80) from Cr, Ni, As, and Hg; 8.2% of the samples presented a considerable risk of contamination (80 ≤ Er < 160) for Cd and Hg; and only 0.6% of the soil samples had a very high risk of contamination (160 ≤ Er < 320) per Hg.

In Julcani, it was also seen that the majority of the samples (86.1%) were considered to be at low risk of contamination (Er < 40); a small percentage (6.9%) of samples were considered to have a moderate contamination risk (40 ≤ Er < 80) from metals As, Cd, Hg, and Pb; 2.8% of samples presented a considerable risk of contamination (80 ≤ Er < 160) for Hg; and 4.2% of soil samples had a high risk of contamination (160 ≤ Er < 320) from As and Hg.

If we consider the individual ecological risk (Er) index, the levels of contamination (from lowest to highest) by heavy metals from the surface horizons of soils were as follows: La Zanja < Julcani < Colquirrumi.

When considering the RI of all the metals studied, the contamination levels were as follows: in La Zanja, all soil samples (100%), present low ecological risk of potential contamination (RI < 150). In Colquirrumi, 38.1% of the samples had a low ecological risk of potential contamination (RI < 150); 57.1% presented a moderate ecological risk potential (150 ≤ RI < 300); and 4.8% faced a significant potential ecological risk (300 ≤ RI < 600). In Julcani, 44.4% of soil samples presented a low ecological risk of potential contamination (RI < 150); 44.4% had a moderate ecological risk of potential contamination (150 ≤ IR < 300); and soils with a considerable risk of contamination (300 ≤ RI <600) were in the minority (11.1%).

Furthermore, when considering the potential ecological risk index (RI), the levels of contamination (from low to high) from heavy metals from the superficial horizons of soils were as follows: La Zanja < Julcani < Colquirrumi.

### 3.4. Spatial Distribution of Heavy Metal Content in Soil

**Figure 5:**
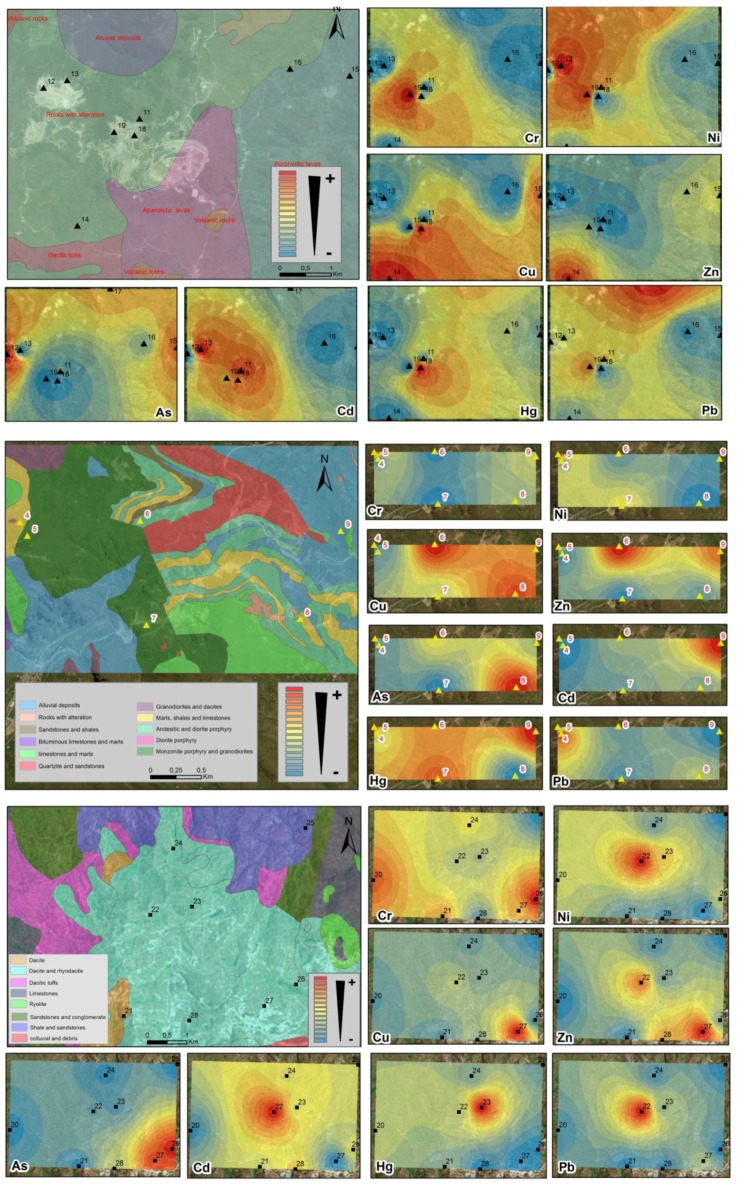
Lithological map-sampling points (soil profiles) and spatial distribution of heavy metals in soils of the Zanja (top), Colquirrumi (middle) and Julcani (bottom).

The use of the geostatistical method of ordinary kriging spatial interpolation allowed us to predict the spatial distribution of the concentrations of each metal in the areas not sampled (Figure 5), based on the values from samples collected in the field campaign, so samples closer to each other presented better correlations and similarity than the more distant ones.

In the area of La Zanja, the distributions of the heavy metals Ni, Cd, and Pb were similar as they had the highest concentrations in the NW and W of the studied area, in the superficial horizons of Profiles 13 and 17–19, which developed on rock alterations (quartz-alunite, quartz-kaolin, etc.) and on a porphyritic rhyolitic dome. These anomalies were located in two current areas of exploitation in Au and Ag mines (in the San Pedro Sur and Pampa Verde fields). The highest concentration of Cu was in the SW quadrant of the zone in Sample 14. The Cr content was abnormally high in the NE quadrant of the zone (Soil 15, developed on porphyritic lavas). The highest concentration of Zn occurred as a concentric circle in the SW quadrant of the studied area in Sample 14, and As dominated in the W-limit of the zone (Soil 12). With regard to Hg content, a geochemical anomaly existed where Profile 18 was sampled.

In the Colquirrumi zone, the Zn and Cd heavy metal distributions were quite similar since they presented the highest concentrations in the NE and N corners of the studied area, in the surface horizons of the Profiles 6 (developed on sandstones and intercalated shales with sills in the Inca Formation) in the case of Zn, and in Profile 9 (developed on limestones and marls of the Pariatambo Formation) in the case of Cd. Furthermore, the distributions of heavy metals As and Hg (given their abnormally high concentrations in the SE quadrant of the studied area) in soils developed on marls, shales and limestones of the Chulec Formation. The highest Pb content was in the NW quadrant in Sample 5 (soil developed on monzonites and granodiorites). The Cu value identified a geochemical anomaly in the form of two concentric circles located in the N (Soil 6) and SE (Soil 8). Ni was present in the SW quadrant (Soils 4 and 7), and finally, the highest concentration of Cr was in the NW quadrant (Soil 4) and in the eastern half of the area (Soils 8 and 9).

In the Julcani zone, the distributions of the heavy metals Ni, Cd, Hg, and Pb were similar and showed a decrease in the form of concentric circles, from the center of the zone towards the periphery (the highest concentrations were Soils 22 and 23, developed on dacitic rocks and riodacites). The contents of Cu, Zn, and As were abnormally high in the SE part of the studied region (Soil samples 27, 26 and 22, developed on dacites and riodacites), which meant that there may have been several contaminations from point sources. The Cr value indicated a higher distribution in the form of two concentric circles located in the SE and E of the investigated area (Soil samples 26, on dacites and riodacites; and 20, on Triassic-Jurassic age limestones).

**Figure 6:**
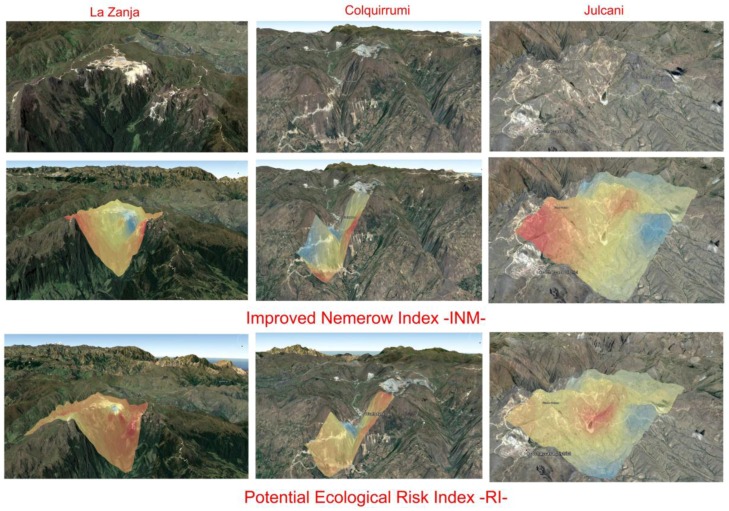
Spatial distribution of the improved Nemerow index (I_IN_) and the potential ecological risk index (RI).

In addition, using both the ordinary kriging interpolation method and ArcGIS 10.4 (Esri), soil contamination rates (I_IN_ and RI) were depicted that reflected the overall pollution situation caused by the simultaneous presence of the eight heavy metals in the three areas studied (Figure 6).

## 4. Conclusions

Soils studied in the Andes Mountains (Peru) were characterized by geochemical baselines with high concentrations of potentially toxic heavy metals. The average concentrations of some elements were much higher than the values obtained from other parts of the world, particularly for Pb, Cu, Zn, As, Cd, and Hg.

Several pollution indices (I_geo_, I_IN_, and RI) have been used as tools to diagnose pollution. Data revealed that the soils were uncontaminated, or slightly contaminated at La Zanja (Cajamarca, Peru); significantly contaminated at Colquirrumi (Cajamarca, Peru); and moderately contaminated in Julcani (Huancavelica, Peru).

If we conducted an environmental risk assessment using the Nemerow index, the soils of La Zanja (virgin zone without the presence of mining operations) have values ranging between contaminated and heavily contaminated soils; however, using the improved Nemerow index I_IN_, most soils fit within Classes 0 and 1 (not contaminated to moderately contaminated). Therefore, there was a great difference in calculating the percentage and the degree of contamination in soils, through the use of different contamination indices. Thus, it is our belief that the I_NM_ was much more accurate in assessing the environmental risks of heavy metal contamination.

The distribution patterns of heavy metal concentrations were mainly influenced by the lithology and geochemistry of the parent rock and by the existence of metallogenic belts in the studied area.

It is our goal to see that the results of this study contribute to the creation or be added to an environmental database of soils in this mining region of the Peruvian Andes, and to facilitate the development of management strategies and remediation of soils contaminated by heavy metals.

## Figures and Tables

**Table 1:** Statistical data of the main properties of the soils studied in La Zanja, Colquirrumi, and Julcani.

Area	Sand (%)	Silt (%)	Clay (%)	pH (H_2_O)	Organic C (%)	CEC cmol·Kg^−1^
**La Zanja *N* = 21**	*M*: 22.9 ± 11.94 *R*: (2.5–47.5)	45.2 ± 10.62 (24.5–65.6)	31.8 ± 14.22 (6.1–58.8)	4.6 ± 0.43 (4.0–5.7)	4.2 ± 4.74 (0.13–15.2)	31.8 ± 14.22 (5.1–68.9)
**Colquirrumi *N* = 20**	29.5 ± 13.76 (6.6–53.6)	41.5 ± 20.16 (24.0–98.96)	30.8 ± 8.52 (17.4–47.8)	4.6 ± 0.85 (3.1–6.8)	5.04 ± 5.69 (0.25–19.7)	31.7 ± 22.49 (8.3–82.1)
**Julcani *N* = 18**	46.3 ± 10.99 (22.4–68.3)	33.4 ± 6.28 (22.2–49.2)	20.3 ± 7.04 (9.4–34.6)	5.4 ± 0.81 (4.8–7.5)	3.86 ± 3.34 (0.23–8.2)	27.8 ± 10.01 (10.0–48.5)

*M*: mean ± standard deviation, *R*: rank. CEC: capacity cation exchange.

**Table 2:** Statistical analysis of heavy metals in the surface horizons of soils.

**La Zanja**	**Cr**	**Ni**	**Cu**	**Zn**	**As**	**Cd**	**Hg**	**Pb**
Mean ($\bar{x}$)	6.10	64.10	17.16	45.94	30.29	2.28	0.30	51.84
Geometric mean	5.33	58.08	10.71	42.41	18.23	2.27	0.21	38.89
Median (Me)	5.21	57.30	12.50	44.00	15.15	2.27	0.15	35.00
Standard deviation (SD)	3.39	30.38	15.05	21.36	29.48	0.10	0.34	48.40
Minimum (min)	2.20	28.40	1.90	26.80	3.00	2.14	0.10	11.60
Maximum (max)	12.07	119.70	35.80	80.20	81.00	2.43	1.05	170.70
Variable coefficient	0.56	0.47	0.88	0.46	0.97	0.04	1.12	0.93
Kurtosis	0.44	−0.23	−2.59	1.52	−0.79	−1.15	5.33	5.39
Skewness	0.92	0.80	0.42	1.23	0.92	0.14	2.28	2.23
Percentil 25	3.56	39.10	3.70	28.15	8.70	2.20	0.12	24.25
Percentil 75	9.19	86.60	32.95	64.70	59.30	2.36	0.37	64.70
Natural Geological Background	3.39	49.84	23.60	44.51	23.25	2.18	0.35	41.40
**Colquirrumi**	**Cr**	**Ni**	**Cu**	**Zn**	**As**	**Cd**	**Hg**	**Pb**
Mean ($\bar{x}$)	16.58	39.94	197.70	1893.06	428.32	13.03	7.97	2068.76
Geometric mean	12.00	22.16	125.64	979.02	253.33	7.44	4.34	1421.09
Median (Me)	14.37	18.30	222.30	2650.85	477.20	17.75	4.00	1296.10
Standard deviation (SD)	13.39	39.31	142.90	1415.80	333.48	9.65	12.23	2033.25
Minimum (min)	1.87	1.10	8.70	29.10	10.00	0.37	1.00	255.60
Maximum (max)	55.05	151.20	509.00	3764.40	1231.10	27.10	57.26	8414.70
Variable coefficient	0.81	0.98	0.72	0.75	0.78	0.74	1.54	0.98
Kurtosis	2.41	1.54	−0.68	−1.82	0.57	−1.83	14.39	4.30
Skewness	1.53	1.26	0.30	−0.20	0.82	−0.13	3.58	2.06
Percentil 25	6.57	7.55	40.55	185.65	141.05	3.18	1.50	983.75
Percentil 75	18.19	64.30	301.15	3087.18	603.80	20.19	9.67	2244.15
Natural Geological Background	2.62	12.05	147.20	165.63	218.10	5.84	7.15	1538.70
**Julcani**	**Cr**	**Ni**	**Cu**	**Zn**	**As**	**Cd**	**Hg**	**Pb**
Mean ($\bar{x}$)	16.34	13.93	11.29	139.36	61.69	5.78	5.95	473.81
Geometric mean	15.62	12.58	9.91	94.90	28.05	5.52	2.99	301.68
Median (Me)	16.40	11.10	9.40	90.40	24.40	5.96	4.60	238.40
Standard deviation (SD)	8.70	7.20	5.00	29.60	3.40	3.12	0.41	95.60
Minimum (min)	24.12	29.00	29.09	358.03	201.40	9.36	22.01	1921.60
Maximum (max)	4.97	7.08	7.19	123.50	77.72	1.82	6.75	577.95
Variable coefficient	0.30	0.51	0.64	0.89	1.26	0.31	1.13	1.22
Kurtosis	−0.69	1.54	5.72	−0.84	0.16	1.51	4.36	6.11
Skewness	0.01	1.28	2.25	0.91	1.35	0.36	1.92	2.40
Percentil 25	12.25	8.31	7.20	38.40	11.35	4.61	0.63	159.15
Percentil 75	20.24	17.90	12.95	265.30	131.09	6.28	8.11	622.63
Natural Geological Background	12.13	9.08	5.13	54.87	10.94	5.84	3.43	195.88
World Mean [41]	50	25	12	40	20	0.40	0.10	15
World Ranks [10]	2–1500	2–500	1–200	1–800	0.1–50	0.01–2	0.01–0.5	2–200
World Ranks [42]	1–1500	1–1500	0.1–250	1–1500	0.1–250	0.01–2	0.01–2	1–1500

**Table 3:** Statistical analysis of heavy metals in all horizons (surface and mother rock) of the soils of La Zanja and the geochemical baseline.

La Zanja	Cr	Ni	Cu	Zn	As	Cd	Hg	Pb
Mean ($\bar{x}$)	5.26	60.13	24.41	52.11	37.82	2.26	0.35	56.03
Standard deviation (S)	3.49	27.43	18.99	31.48	38.03	0.12	0.31	48.20
Median (Me)	3.88	57.30	13.10	45.05	18.80	2.23	0.22	35.95
Geometric mean	4.20	53.59	16.48	43.62	20.86	2.26	0.25	40.25
Standard deviation geometric	2.03	1.69	2.86	1.90	3.32	1.05	2.32	2.31
Minimum (min)	0.97	14.40	1.90	13.50	3.00	2.10	0.10	11.10
Maximum (max)	12.07	119.70	52.40	106.50	111.60	2.50	1.05	170.70
Percentile 25	2.52	40.70	9.80	27.48	8.18	2.16	0.12	19.82
Percentile 75	8.46	81.30	45.90	77.85	68.02	2.36	0.60	86.45
Proposal of baseline values	8.26	56.97	22.20	47.42	27.50	4.36	4.89	44.87

**Table 4:** Indexes of geo-accumulation (I_geo_) of heavy metals and improved Nemerow index (I_IN_).

**La Zanja**	**I_geo_**								**I_geo_ave**	**I_geo_max**	**I_IN_**
	**Cr**	**Ni**	**Cu**	**Zn**	**As**	**Cd**	**Hg**	**Pb**			
Mean ($\bar{x}$)	0.07	−0.36	−1.73	−0.65	−1.10	−0.52	−1.80	−0.68	−0.72	0.68	0.80
Median (Me)	0.03	−0.38	−1.50	−0.60	−1.44	−0.53	−2.29	−0.83	−0.77	0.69	0.83
Minimum (min)	−1.21	−1.40	−4.22	−1.32	−3.70	−0.61	−2.88	−2.42	−1.28	−0.07	0.33
Maximum (max)	1.25	0.68	0.02	0.26	1.05	−0.43	0.51	1.46	−0.19	1.46	1.05
Standard deviation (SD)	0.79	0.68	1.76	0.63	1.65	0.06	1.21	1.11	0.41	0.51	0.24
**Colquirrumi**	**I_geo_**								**I_geo_ave**	**I_geo_max**	**I_IN_**
	**Cr**	**Ni**	**Cu**	**Zn**	**As**	**Cd**	**Hg**	**Pb**			
Mean ($\bar{x}$)	1.61	0.29	−0.81	1.98	−0.37	−0.24	−1.31	−0.70	0.00	3.02	2.21
Median (Me)	1.87	0.02	0.01	3.42	0.54	1.02	−1.42	−0.83	0.28	3.41	2.46
Minimum (min)	−1.07	−4.04	−4.67	−3.09	−5.03	−4.57	−3.42	−3.17	−1.96	1.81	1.39
Maximum (max)	3.81	3.06	1.20	3.92	1.91	1.63	2.42	1.87	0.86	3.92	2.79
Standard deviation (SD)	1.28	1.80	1.71	2.22	1.93	1.99	1.53	1.30	0.77	0.78	0.50
**Julcani**	**I_geo_**								**I_geo_ave**	**I_geo_max**	**I_IN_**
	**Cr**	**Ni**	**Cu**	**Zn**	**As**	**Cd**	**Hg**	**Pb**			
Mean ($\bar{x}$)	−0.22	−0.12	0.36	0.21	0.77	−0.67	−0.78	0.04	−0.05	1.64	1.36
Median (Me)	−0.15	−0.30	0.29	0.14	0.57	−0.56	−0.16	−0.30	−0.14	2.10	1.52
Minimum (min)	−1.06	−0.92	−0.62	−1.48	−2.27	−1.49	−3.67	−1.62	−1.15	−0.58	0.27
Maximum (max)	0.41	1.09	1.92	2.12	3.62	0.10	2.10	2.71	0.94	3.62	2.56
Standard deviation (SD)	0.47	0.68	0.73	1.38	2.00	0.48	2.02	1.34	0.59	1.53	0.89

**Table 5:** Ecological individual risk (E_r_^i^) and potential ecological risk index (RI) of the soils studied.

**La Zanja**	**Er Cr**	**Er Ni**	**Er Cu**	**Er Zn**	**Er As**	**Er Cd**	**Er Hg**	**Er Pb**	**RI**
Mean ($\bar{x}$)	3.60	6.43	3.64	1.03	11.65	31.32	24.84	6.26	75.38
Median (Me)	3.07	5.75	2.65	0.99	5.83	31.17	12.24	4.23	65.30
Minimum (min)	1.30	2.85	0.40	0.60	1.15	29.45	8.16	1.40	53.75
Maximum (max)	7.12	12.01	7.58	1.80	31.15	33.44	85.71	20.62	130.45
Standard deviation (S)	2.00	3.05	3.19	0.48	11.34	1.37	27.90	5.85	24.70
**Colquirrumi**	**Er Cr**	**Er Ni**	**Er Cu**	**Er Zn**	**Er As**	**Er Cd**	**Er Hg**	**Er Pb**	**RI**
Mean ($\bar{x}$)	12.66	16.57	6.72	11.43	19.64	66.93	44.57	6.72	167.35
Median (Me)	10.97	7.59	7.55	16.00	21.88	91.16	22.38	4.21	175.31
Minimum (min)	1.43	0.46	0.30	0.18	0.46	1.90	5.59	0.83	51.12
Maximum (max)	42.02	62.74	17.29	22.73	56.45	139.21	320.34	27.34	362.41
Standard deviation (S)	10.22	16.31	4.85	8.55	15.29	49.59	68.42	6.61	65.95
**Julcani**	**Er Cr**	**Er Ni**	**Er Cu**	**Er Zn**	**Er As**	**Er Cd**	**Er Hg**	**Er Pb**	**RI**
Mean ($\bar{x}$)	2.69	7.67	11.00	2.54	56.39	29.71	69.41	12.09	191.51
Median (Me)	2.70	6.11	9.16	1.65	22.30	30.62	53.64	6.09	154.82
Minimum (min)	1.43	3.96	4.87	0.54	3.11	16.05	4.73	2.44	84.03
Maximum (max)	3.98	15.97	28.35	6.53	184.10	48.08	256.68	49.05	348.17
Standard deviation (S)	0.82	3.90	7.01	2.25	71.04	9.33	78.71	14.75	85.80

## Supplementary Materials

The following are available online at http://www.mdpi.com/1660-4601/14/8/859/s1, Nemerow Index Kmz, RI Index Kmz.
